# Quantum-Solving Algorithm for d’Alembert Solutions of the Wave Equation

**DOI:** 10.3390/e25010062

**Published:** 2022-12-29

**Authors:** Yuanye Zhu

**Affiliations:** 1 Center on Frontiers of Computing Studies and School of Computer Science, Peking University, Beijing 100871, China; yuanyezhu@pku.edu.cn; 2 State Key Laboratory of Low-Dimensional Quantum Physics and Department of Physics, Tsinghua University, Beijing 100084, China; zhuyy16@tsinghua.org.cn

**Keywords:** quantum algorithm, quantum computation, quantum information

## Abstract

When faced with a quantum-solving problem for partial differential equations, people usually transform such problems into Hamiltonian simulation problems or quantum-solving problems for linear equation systems. In this paper, we propose a third approach to solving partial differential equations that differs from the two approaches. By using the duality quantum algorithm, we construct a quantum-solving algorithm for solving the first-order wave equation, which represents a typical class of partial differential equations. Numerical results of the quantum circuit have high precision consistency with the theoretical d’Alembert solution. Then the routine is applied to the wave equation with either a dissipation or dispersion term. As shown by complexity analysis for all these cases of the wave equation, our algorithm has a quadratic acceleration for each iteration compared to the classical algorithm.

## 1. Introduction

Most scientific problems can be solved by studying the laws governing the evolution of physical quantities in space and time. Therefore, partial differential equations undoubtedly play an extremely important role in the field of natural sciences. However, the problem of solving partial differential equations is extremely difficult. While if quantum algorithms are introduced and the problems of partial differential equations are solved on a quantum computer, it can achieve accelerated characteristics compared to classical algorithms.

The usual quantum algorithm for solving partial differential equations proceeds as follows. First, discretize the space so that the function $f\left(x,t\right)$ becomes a vector $f\left(t\right)$ and map its normalized components to the quantum state components, i.e., $|x\left(t\right)\rangle =\sum_{i}f_{i}^{\prime }\left(t\right)|x_{i}\rangle$, where $f_{i}^{\prime }\left(t\right)$ is the *i*-th component of the vector $f\left(t\right)$ after normalization. Next, the vectors encoded onto the quantum states are mapped onto a fixed model. Most quantum algorithms for solving partial differential equations rely on Hamiltonian simulations [1,2,3,4] or a linear equation system-solving algorithm (HHL algorithm) [5].

In the following, the main ideas of the above two solution methods will be briefly reviewed with examples. The solution method based on Hamiltonian simulation [6,7,8] that maps partial differential equations to the Schrödinger equation will be introduced first. This method maps equations with a similar structure to the Schrödinger equation to the Schrödinger equation and transforms the equation solving problem into a Hamiltonian simulation problem. For example, solving the Black–Scholes equation [9]

(1) $$\frac{\partial f}{\partial t}=af+b\frac{\partial f}{\partial x}-c\frac{\partial^{2}f}{\partial x^{2}}.$$

The equation can be written in the following form

(2) $$\frac{\partial f}{\partial t}=Af.$$

It is obvious that the equation is formally similar to the Schrödinger equation. Thus the *A* operator can be mapped to the Hamiltonian in Schrödinger’s equation in such a way that $A=ib\hat{p}+\left(aI+c{\hat{p}}^{2}\right),\hat{p}=-i\partial_{x}$. One can split *A* into Hermitian and anti-Hermitian parts, i.e., $A=A_{H}+A_{aH}$, where

(3) $$A_{aH}=ib\hat{p},\;A_{H}=aI+c{\hat{p}}^{2}.$$

The vector $f\left(t\right)$, obtained by discretizing the function, is encoded onto the state vector |x(ϵ)〉, using the Trotter product formula

(4) $$|x\left(\epsilon \right)\rangle =e^{A\epsilon }|x_{0}\rangle \approx e^{A_{H}\epsilon }e^{A_{aH}\epsilon }|x_{0}\rangle .$$

The problem of solving the partial differential equation is transformed into the problem of a Hamiltonian simulation. The process of simulating the action of the above Hamiltonian, i.e., the quantum state $|x_{0}\rangle$, evolves under the designed Hamiltonian to obtain the final state. The solution of the original equation at different moments can be obtained by measuring the final state for different iterations.

In fact, it is efficient to use a Hamiltonian simulation to construct quantum algorithms for solving partial differential equations, which can solve first-order partial differential equations (requiring that the Hermitian and anti-Hermitian parts of the matrix *A* decomposition commute with each other) and second-order partial differential equations such as the wave equation. However, not all partial differential equations have the algebraic structure of Schrödinger’s equation. The quantum algorithm [7,10,11,12] for solving partial differential equations is presented below using the HHL algorithm. For the partial differential equation with the following structure after spatial discretization

(5) $$\dot{x}=Ax+b.$$

Using Euler’s method to discretize time gives

(6) $$\frac{x\left(t_{j+1}\right)-x\left(t_{j}\right)}{h}\approx Ax\left(t_{j}\right)+b.$$

Let $x_{j}=x\left(t_{j}\right)$, the partial differential equation can be transformed into the following linear equation system; as an example, only the result of j≤2 is given here,

(7) $$\left[\begin{array}{ccc} I & 0 & 0\\ -(I+Ah) & I & 0\\ 0 & -(I+Ah) & I \end{array}\right]\left[\begin{array}{c} x_{0}\\ x_{1}\\ x_{2} \end{array}\right]=\left[\begin{array}{c} x_{\mathrm{in}}\\ bh\\ bh \end{array}\right].$$

This system of equations is then solved using the HHL algorithm to obtain the following quantum states

(8) $$|x\rangle =\sum_{j=0}^{N_{t}}|t_{j}\rangle |x_{j}\rangle .$$

The quantum state contains the solution of the partial differential equation $t_{0}$ to $t_{j}$ moments. Therefore, are there any other efficient algorithms for solving partial differential equations besides the above two methods? In this article, a third method different from the above two, the duality quantum algorithm [13,14,15,16] with amplitude amplification [17,18,19,20], is used to construct a quantum algorithm for solving the partial differential equation. The duality quantum algorithm also brings a speed-up effect compared to the classical algorithm [21,22,23].

This paper is organized as follows. First of all, the duality quantum algorithm will be used to solve the first-order wave equation with the d’Alembert solution. In the second and third parts of this paper, we will use the duality quantum algorithm to construct a solution algorithm to the wave equations with dissipation and dispersion terms. In these three parts of the paper, for these three problems, we use numerical simulations and present the results of the quantum algorithm solutions in the form of pictures for comparison with the theoretical values. At the end of this paper, we will analyze the complexity of our algorithm for solving the wave equations.

## 2. Duality Quantum Algorithm for Solving the First-Order Wave Equation

When talking about the wave equation, people must first think of the second-order linear hyperbolic type equation

(9) $$\frac{\partial^{2}u}{\partial t^{2}}+k^{2}\frac{\partial^{2}u}{\partial x^{2}}=0.$$

The general solution can be written as $u\left(x,t\right)=f\left(x-kt\right)+g\left(x+kt\right)$, where *f*, *g* are two arbitrary functions. $f\left(x-kt\right)$ and $g\left(x+kt\right)$ represent waves passing along the *x*-axis with constant velocity to the right and to the left. Since Equation (9) is a linear homogeneous equations, its solutions are superposed. Therefore, *f* and *g* are two traveling waves that propagate independently without interfering with each other. If one focuses on only one of these two waves, Equation (9) degenerates to a linear hyperbolic equation of the first-order

(10) $$\frac{\partial u}{\partial t}+k\frac{\partial u}{\partial x}=0.$$

In this paper, we discretize the continuous independent variable *x* into *N* points, i.e., $x=\left(x_{0},x_{1},\cdots ,x_{N-1}\right)$. Then the spatial part of the function $u\left(x,t_{j}\right)$ at the moment $t_{j}$ is discretized into the vector

(11) $$u\left(x,t_{j}\right)=\left(u\left(x_{0},t_{j}\right),u\left(x_{1},t_{j}\right),\cdots ,u\left(x_{N-1},t_{j}\right)\right),$$

encode it onto the computational basis and define the quantum state ${|\psi \rangle }_{j}$ as

(12) $${|\psi \rangle }_{j}=\frac{\sum_{i=0}^{N-1}u\left(x_{i},t_{j}\right)|i\rangle }{\sqrt{\sum_{i=0}^{N-1}u^{2}\left(x_{i},t_{j}\right)}}.$$

In the following, we will give the quantum algorithm for solving Equation (10) based on the non-unitary evolution of the quantum system. First, the Taylor expansion for each order partial differential term of Equation (10) is

(13) $$\begin{array}{cc} \; & \frac{\partial u}{\partial t}=\frac{u\left(x,t+\tau \right)-u\left(x,t\right)}{\tau }+o\left(\tau \right),\\ \;\; & \frac{\partial u}{\partial x}=\frac{u\left(x+h,t\right)-u\left(x,t\right)}{h}+o\left(h\right). \end{array}$$

Pluging Equation (13) into Equation (10), the difference equation form of Equation (10) is obtained as

(14) $$\frac{u\left(x_{i},t_{j+1}\right)-u\left(x_{i},t_{j}\right)}{\tau }+k\frac{u\left(x_{i+1},t_{j}\right)-u\left(x_{i},t_{j}\right)}{h}=0.$$

Its local truncation error is o(τ+h). When τ, h→0, Equation (14) approximates the original Equation (10). Organizing Equation (14) leads to

(15) $$u\left(x_{i},t_{j+1}\right)=\frac{\tau k\left[u\left(x_{i},t_{j}\right)-u\left(x_{i+1},t_{j}\right)\right]}{h}+u\left(x_{i},t_{j}\right)$$

Let $\Delta =\frac{\tau }{h}$, then the following iterative relation can be obtained from Equation (15).

(16) $$u\left(x_{i},t_{j+1}\right)=\left(1+\Delta k\right)u\left(x_{i},t_{j}\right)-\Delta ku\left(x_{i+1},t_{j}\right)$$

Taking the periodic boundary condition that $u\left(x_{N},t\right)=\left(x_{0},t\right)$, the equation describing the whole system can be written in the following form

(17) $$\left[\begin{array}{c} u\left(x_{0},t_{j+1}\right)\\ \;u\left(x_{1},t_{j+1}\right)\\ \;\ldots \\ u\left(x_{N-1},t_{j+1}\right) \end{array}\right]=A\left[\begin{array}{c} u\left(x_{0},t_{j}\right)\\ \;u\left(x_{1},t_{j}\right)\\ \ldots \\ \;u\left(x_{N-1},t_{j}\right) \end{array}\right]$$

where

(18) $$A=\left[\begin{array}{ccccccc} 1+\Delta k & -\Delta k & 0 & \; & \ldots & \; & 0\\ \;0 & 1+\Delta k & -\Delta k & 0 & \ldots & \; & 0\\ \;\; & \ldots & \; & & \; & & \\ -\Delta k & 0 & \; & & \ldots & 0 & 1+\Delta k \end{array}\right]$$

Then the state ${|\psi \rangle }_{j+1}$ of the system at the next moment, i.e., the moment $t_{j+1}$, can be expressed as ${A|\psi \rangle }_{j}$. It is obvious that the *A*-matrix is not an unitary matrix, so there is no way to achieve it directly by the product of quantum logic gates. Instead, the *A*-matrix has to be split into linear combinations of the unitary operators by the duality model of quantum computation, i.e., $A=\left(1+\Delta k\right)A_{0}-\Delta kA_{1}$, where $A_{0}$ is a unitary matrix of order *N* and

(19) $$A_{1}={\left[\begin{array}{ccccc} 0 & 1 & 0 & \; & 0\\ \;0 & 0 & 1 & \ldots & 0\\ \;\; & \cdots & \; & & \\ \;0 & 0 & 0 & \; & 1\\ \;1 & 0 & 0 & 0 & 0 \end{array}\right]}_{N⊗N}.$$

**Figure 1:**
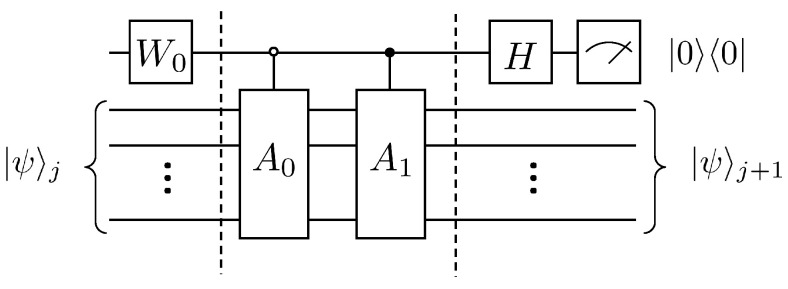
Quantum circuit for solving the first-order wave equation.

By introducing an auxiliary qubit, the operation of the linear combination of unitary operators can be realized and thus equivalently the non-unitary evolution, i.e., ${A|\psi \rangle }_{j}$. Its quantum circuit is shown in Figure 1.

**Figure 2:**
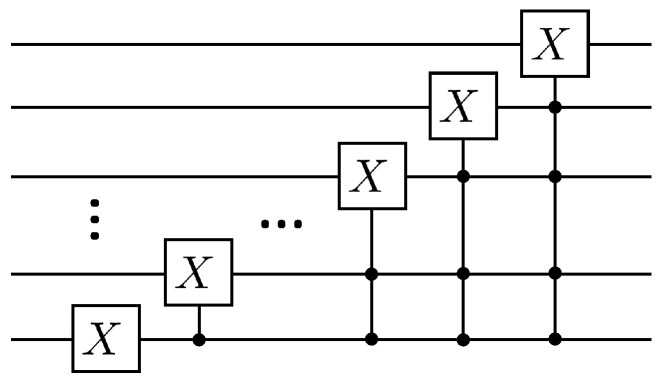
Quantum circuit for realization $A_{1}$ operation.

Where the matrix $A_{1}$ can be decomposed into $C^{n}\left(X\right)$ gates as well as *X* gates with $O\left(\log_{2}N\right)$. The specific quantum circuit that implements the $A_{1}$ operation is shown in Figure 2.

According to Lemma 5.5 and Lemma 7.1 in article [24], a total of $4N-5\log_{2}N-4$ CNOT gates and single-qubit rotating gates are needed if the $A_{1}$ operation continues to be disassembled. The following will explain the duality quantum algorithm for the solution of the first-order wave equation according to Figure 1, where first the auxiliary qubit passes through the $W_{0}$ gate, which has the following effect

(20) $$W_{0}:|0\rangle \to \frac{\left(1+\Delta k\right)|0\rangle -\Delta k|1\rangle }{\sqrt{{\left(1+\Delta k\right)}^{2}+{\left(\Delta k\right)}^{2}}}.$$

Next, after two controlled quantum gates $\left|0\rangle \langle 0\right|⊗A_{0}$ and $\left|1\rangle \langle 1\right|⊗A_{1}$, the quantum state evolves as

(21) $$\frac{\left(1+\Delta k\right)|0\rangle A_{0}{|\psi \rangle }_{j}-\Delta k|1\rangle A_{1}{|\psi \rangle }_{j}}{\sqrt{{\left(1+\Delta k\right)}^{2}+{\left(\Delta k\right)}^{2}}}.$$

Then the quantum state after the Hadamard transformation is

(22) $$\frac{1}{\sqrt{2}}|0\rangle \left[\frac{\left(1+\Delta k\right)A_{0}{|\psi \rangle }_{j}-\Delta kA_{1}{|\psi \rangle }_{j}}{\sqrt{{\left(1+\Delta k\right)}^{2}+{\left(\Delta k\right)}^{2}}}\right]+\frac{1}{\sqrt{2}}|1\rangle \left[\frac{\left(1+\Delta k\right)A_{0}{|\psi \rangle }_{j}+\Delta kA_{1}{|\psi \rangle }_{j}}{\sqrt{{\left(1+\Delta k\right)}^{2}+{\left(\Delta k\right)}^{2}}}\right].$$

Finally, after the measurement to select the state of the auxiliary qubit as 0, the state of the working qubits at this time is ${|\psi \rangle }_{j+1}$, that is

(23) $$\frac{\left(1+\Delta k\right)A_{0}{|\psi \rangle }_{j}-\Delta kA_{1}{|\psi \rangle }_{j}}{\sqrt{{\left(1+\Delta k\right)}^{2}+{\left(\Delta k\right)}^{2}}}.$$

Define the coefficients $C_{j}$ as

(24) $$\sqrt{\sum_{i=0}^{N-1}u^{2}\left(x_{i},t_{j}\right)\left[{\left(1+\Delta k\right)}^{2}+{\left(\Delta k\right)}^{2}\right]}.$$

The amplitude under the computational basis of the quantum state ${|\psi \rangle }_{j+1}$ is enlarged by a factor of $C_{j}$ to obtain the column vector $u(x_{i},t_{j+1})$, which is the state of the system at the moment $t_{j+1}$. The analysis yields that the computational complexity of this algorithm is $O\left(N\right)$ per iteration, while the complexity of the classical algorithm is $O\left(N^{2}\right)$. The specific calculation of the complexity is presented at the end of this paper.

The following equation will be used as an example

(25) $$\left\{\begin{array}{c} \frac{\partial u}{\partial t}+2\frac{\partial u}{\partial x}=0\\ \;u\left(x,0\right)=-\sin2\pi x+\frac{\sin\;4\pi x}{2}-\frac{\sin\;6\pi x}{3} \end{array}\right.$$

to show the duality quantum algorithm for the solution of the first-order wave equation. First of all, one period of the function, i.e., [0,1], is chosen, and this interval is discretized into 32 points, i.e., h=0.03125 in Equation (15). Thus, the function value of 32 discrete points can be encoded using 5 qubits. Choose Δ=0.1 in Equation (16). Then τ=0.003125, which represents the time interval for each iteration of the system evolution. According to Equation (20), the effect of the action of $W_{0}$ can be determined as

(26) $$W_{0}:|0\rangle \to \frac{6\sqrt{37}}{37}|0\rangle -\frac{\sqrt{37}}{37}|1\rangle$$

**Figure 3:**
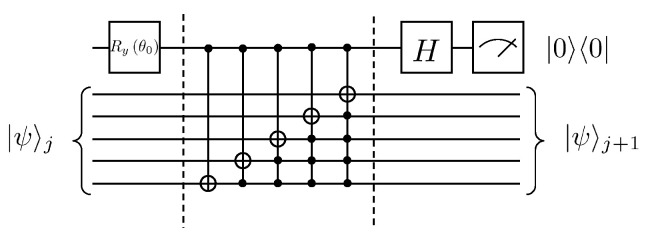
Example of quantum circuit for solving the first-order wave equation, where $\theta_{0}=-0.33$.

This gives $W_{0}$ as $R_{y}\left(-0.33\right)$. Thus far, the quantum circuit for each iteration of the solution to Equation (25) can be given in Figure 3.

**Figure 4:**
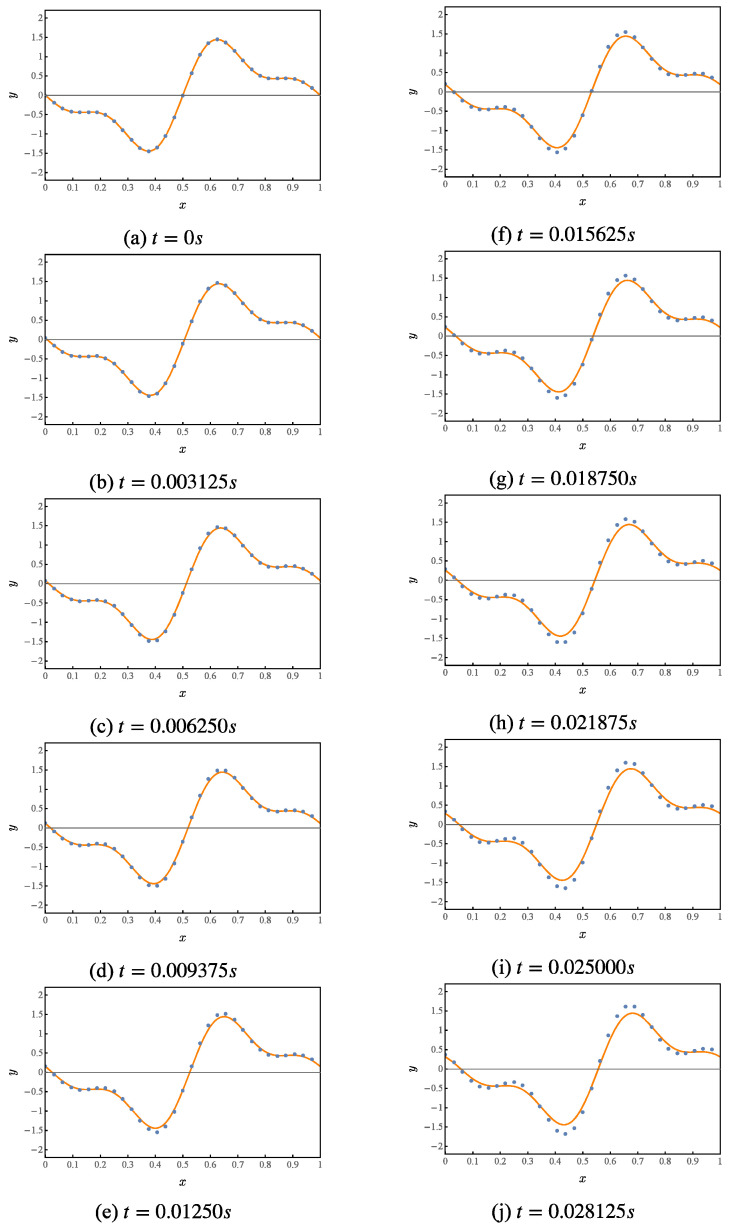
Numerical simulation of a quantum solution algorithm for the first-order wave equation.

Numerical simulation of the first 10 iterations of this quantum circuit, whose results are shown in Figure 4. Where the orange curve represents the theoretical value. The blue points represent the results given by the numerical simulation of the quantum solution algorithm.

## 3. Duality Quantum Algorithm for the Solution of the Traveling Wave Dissipation Problem

By adding the dissipation term to Equation (10), the traveling wave equation with dissipation is obtained as

(27) $$\frac{\partial u}{\partial t}+k\frac{\partial u}{\partial x}-\alpha \frac{\partial^{2}u}{\partial x^{2}}=0.$$

The Taylor expansion for each term of Equation (27) is

(28) $$\begin{array}{cc} \frac{\partial u}{\partial t} & =\frac{u\left(x,t+\tau \right)-u\left(x,t\right)}{\tau }+o\left(\tau \right),\\ \;\frac{\partial u}{\partial x} & =\frac{u\left(x+h,t\right)-u\left(x,t\right)}{h}+o\left(h\right),\\ \;\frac{\partial^{2}u}{\partial x^{2}} & =\frac{u(x-h,t)-2u(x,t)+u(x+h,t)}{h^{2}}+o\left(h^{2}\right). \end{array}$$

Differentiating Equation (27) yields

(29) $$\begin{array}{cc} \frac{u\left(x_{i},t_{j+1}\right)-u\left(x_{i},t_{j}\right)}{\tau }+ & k\frac{u\left(x_{i+1},t_{j}\right)-u\left(x_{i},t_{j}\right)}{h}\\ - & \alpha \frac{u\left(x_{i-1},t_{j}\right)-2u\left(x_{i},t_{j}\right)+u\left(x_{i+1},t_{j}\right)}{h^{2}}=0. \end{array}$$

The collation leads to

(30) $$\begin{array}{cc} u\left(x_{i},t_{j+1}\right)= & \tau k\frac{u\left(x_{i},t_{j}\right)-u\left(x_{i+1},t_{j}\right)}{h}\\ \; & +\tau \alpha \frac{u\left(x_{i-1},t_{j}\right)-2u\left(x_{i},t_{j}\right)+u\left(x_{i+1},t_{j}\right)}{h^{2}}+u\left(x_{i},t_{j}\right). \end{array}$$

Let $\Delta =\frac{\tau }{h}$ and $\Delta_{1}=\alpha \frac{\Delta }{h}$, then the following iterative relation can be obtained

(31) $$u\left(x_{i},t_{j+1}\right)=\Delta_{1}u\left(x_{i-1},t_{j}\right)+\left(k\Delta -2\Delta_{1}+1\right)u\left(x_{i},t_{j}\right)+\left(\Delta_{1}-k\Delta \right)u\left(x_{i+1},t_{j}\right).$$

Take the periodic boundary condition that $u\left(x_{N},t\right)=u\left(x_{0},t\right)$. Then, the equation describing the whole system can be written in the following form

(32) $$\left[\begin{array}{c} u\left(x_{0},t_{j+1}\right)\\ u\left(x_{1},t_{j+1}\right)\\ \ldots \\ u\left(x_{N-1},t_{j+1}\right) \end{array}\right]=A\left[\begin{array}{c} u\left(x_{0},t_{j}\right)\\ u\left(x_{1},t_{j}\right)\\ \ldots \\ u\left(x_{N-1},t_{j}\right) \end{array}\right].$$

In Equation (32)

(33) $$A=\left[\begin{array}{ccccccccc} a & b & 0 & \; & \ldots & \; & & 0 & c\\ c & a & b & 0 & \ldots & \; & & \; & 0\\ \; & \ldots & \; & & \; & & \; & \\ 0 & \; & & \; & \ldots & 0 & c & a & b\\ b & 0 & \; & & \ldots & \; & 0 & c & a \end{array}\right],$$

in which

(34) $$\begin{array}{cc} \; & a=k\Delta -2\Delta_{1}+1,\\ \; & b=\Delta_{1}-k\Delta ,\\ \; & c=\Delta_{1}. \end{array}$$

**Figure 5:**
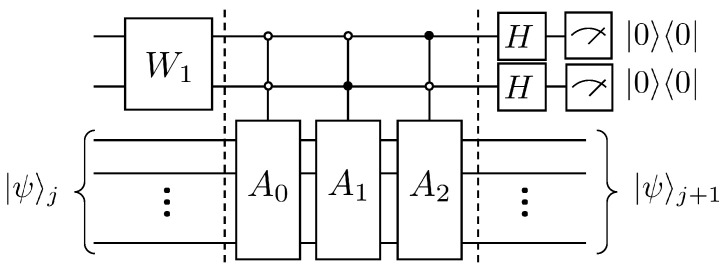
Quantum circuit for solving the dissipation problem of the first-order wave equation.

Following the encoding method of Equation (12), then the state ${|\psi \rangle }_{j+1}$ of the system at the next moment, i.e., the moment $t_{j+1}$, can be expressed as ${A|\psi \rangle }_{j}$. The matrix $A=\left(k\Delta -2\Delta_{1}+1\right)A_{0}+\left(\Delta_{1}-k\Delta \right)A_{1}+\Delta_{1}A_{2}$, where $A_{2}=A_{1}^{†}$. Thus the operation of a linear combination of unitary operators can be equivalently implemented by introducing two auxiliary qubits, whose quantum circuit is shown in Figure 5.

In the following, the duality quantum algorithm for solving the dissipation problem of the first-order wave equation is explained in conjunction with the quantum circuit (Figure 5), where the first the auxiliary qubits pass through the $W_{1}$ gate, which has the following effect

(35) $$W_{1}:|00\rangle \to \frac{\left(k\Delta -2\Delta_{1}+1\right)|00\rangle +\left(\Delta_{1}-k\Delta \right)|01\rangle +\Delta_{1}|10\rangle }{\sqrt{{\left(k\Delta -2\Delta_{1}+1\right)}^{2}+{\left(\Delta_{1}-k\Delta \right)}^{2}+\Delta_{1}^{2}}}.$$

Next, after three controlled quantum gates $\left|00\rangle \langle 00\right|⊗A_{0}$, $\left|01\rangle \langle 01\right|⊗A_{1}$ and $\left|10\rangle \langle 10\right|⊗A_{2}$ the quantum state evolves as

(36) $$\frac{\left(k\Delta -2\Delta_{1}+1\right)|00\rangle A_{0}{|\psi \rangle }_{j}+\left(\Delta_{1}-k\Delta \right)|01\rangle A_{1}{|\psi \rangle }_{j}+\Delta_{1}|10\rangle A_{2}{|\psi \rangle }_{j}}{\sqrt{{\left(k\Delta -2\Delta_{1}+1\right)}^{2}+{\left(\Delta_{1}-k\Delta \right)}^{2}+\Delta_{1}^{2}}}.$$

Then, the quantum state evolves after the Hadamard transformation of two auxiliary qubits as

(37) $$\begin{array}{cc} \frac{1}{2} & |00\rangle \left[\frac{\left(k\Delta -2\Delta_{1}+1\right)A_{0}{|\psi \rangle }_{j}+\left(\Delta_{1}-k\Delta \right)A_{1}{|\psi \rangle }_{j}+\Delta_{1}A_{2}{|\psi \rangle }_{j}}{\sqrt{{\left(k\Delta -2\Delta_{1}+1\right)}^{2}+{\left(\Delta_{1}-k\Delta \right)}^{2}+\Delta_{1}^{2}}}\right]\\ \; & +\frac{1}{2}|01\rangle \left[\frac{\left(k\Delta -2\Delta_{1}+1\right)A_{0}{|\psi \rangle }_{j}-\left(\Delta_{1}-k\Delta \right)A_{1}{|\psi \rangle }_{j}+\Delta_{1}A_{2}{|\psi \rangle }_{j}}{\sqrt{{\left(k\Delta -2\Delta_{1}+1\right)}^{2}+{\left(\Delta_{1}-k\Delta \right)}^{2}+\Delta_{1}^{2}}}\right]\\ \; & +\frac{1}{2}|10\rangle \left[\frac{\left(k\Delta -2\Delta_{1}+1\right)A_{0}{|\psi \rangle }_{j}+\left(\Delta_{1}-k\Delta \right)A_{1}{|\psi \rangle }_{j}-\Delta_{1}A_{2}{|\psi \rangle }_{j}}{\sqrt{{\left(k\Delta -2\Delta_{1}+1\right)}^{2}+{\left(\Delta_{1}-k\Delta \right)}^{2}+\Delta_{1}^{2}}}\right]\\ \; & +\frac{1}{2}|11\rangle \left[\frac{\left(k\Delta -2\Delta_{1}+1\right)A_{0}{|\psi \rangle }_{j}-\left(\Delta_{1}-k\Delta \right)A_{1}{|\psi \rangle }_{j}-\Delta_{1}A_{2}{|\psi \rangle }_{j}}{\sqrt{{\left(k\Delta -2\Delta_{1}+1\right)}^{2}+{\left(\Delta_{1}-k\Delta \right)}^{2}+\Delta_{1}^{2}}}\right]. \end{array}$$

Finally, the auxiliary qubits are measured to select the state of the auxiliary qubit as 00; then the state of the working qubit at this time is ${|\psi \rangle }_{j+1}$, that is

(38) $$\frac{\left(k\Delta -2\Delta_{1}+1\right)A_{0}{|\psi \rangle }_{j}+\left(\Delta_{1}-k\Delta \right)A_{1}{|\psi \rangle }_{j}+\Delta_{1}A_{2}{|\psi \rangle }_{j}}{\sqrt{{\left(k\Delta -2\Delta_{1}+1\right)}^{2}+{\left(\Delta_{1}-k\Delta \right)}^{2}+\Delta_{1}^{2}}}.$$

Define the coefficient $C_{j}$ as

(39) $$\sqrt{\sum_{i=0}^{N-1}u^{2}\left(x_{i},t_{j}\right)\left[{\left(k\Delta -2\Delta_{1}+1\right)}^{2}+{\left(\Delta_{1}-k\Delta \right)}^{2}+\Delta_{1}^{2}\right]}.$$

The column vector $u(x_{i},t_{j+1})$, which is the state of the system at $t_{j+1}$ moments, is obtained by amplifying the amplitude under the computational basis of the quantum state ${|\psi \rangle }_{j+1}$ by a factor of $C_{j}$.

According to Lemma 5.5 and Lemma 7.1 in article [24] and combined with Figure 5, the computational complexity of this algorithm per iteration is $O\left(N\right)$, while the complexity of the classical algorithm is $O\left(N^{2}\right)$. Thus, the present algorithm has the property of speeding up in each iteration compared to the classical algorithm. The specific calculation of the complexity is presented at the end of this paper.

The following equation will be used as an example

(40) $$\left\{ \begin{array}{c} \frac{\partial u}{\partial t}+2\frac{\partial u}{\partial x}-0.1\frac{\partial^{2}u}{\partial x^{2}}=0\\ u\left(x,0\right)=-\sin\;2\pi x+\frac{\sin\;4\pi x}{2}-\frac{\sin\;6\pi x}{3} \end{array}\right.$$

to show the duality quantum algorithm for the solution of the dissipation problem of the first-order wave equation. First, one period of the function is chosen, i.e., [0,1], and this interval is discretized into 32 points, i.e., h=0.03125 in Equation (28). Thus, the function value of 32 discrete points can be encoded using 5 qubits. Choose Δ=0.2 and $\Delta_{1}=1.28$ in Equation (31). Then τ=0.00625, which represents the time interval for each iteration of system evolution. According to Equation (35), the effect of the action of $W_{1}$ can be determined as

(41) $$W_{1}:|00\rangle \to -\frac{\sqrt{29}}{9}|00\rangle +\frac{22\sqrt{29}}{261}|01\rangle +\frac{32\sqrt{29}}{261}|10\rangle .$$

**Figure A1:**
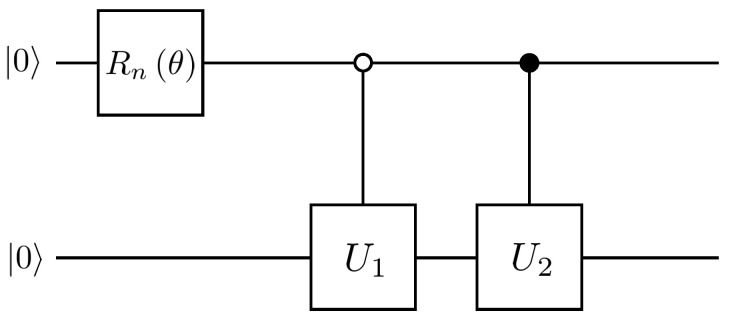
Preparation of two-qubit arbitrary quantum states.

It is constructed as shown in Figure A1 with Equations (A1) and (A2). The revolving gate $R_{n}\left(\theta \right)$ of the first auxiliary qubit is constructed according to Equation (A1), such that

(42) $$R_{n}\left(\theta \right)|00\rangle \to \left(\frac{5\sqrt{1537}}{261}|0\rangle +\frac{32\sqrt{29}}{261}|1\rangle \right)|0\rangle .$$

It is obtained that $R_{n}\left(\theta \right)$ is $R_{y}\left(1.442\right)$. According to Equation (A3), the controlled operator $U_{1}$ is to achieve the following action

(43) $$U_{1}|0\rangle \to \frac{29\sqrt{53}}{265}|0\rangle +\frac{22\sqrt{53}}{265}|1\rangle .$$

**Figure 6:**
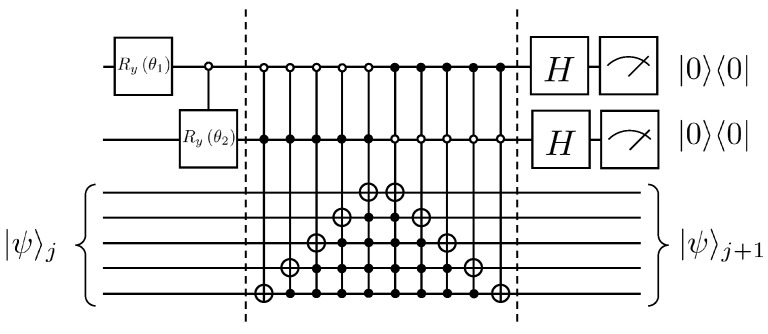
Example of a quantum circuit for solving the dissipation problem of the first-order wave equation, where $\theta_{1}=1.442$, $\theta_{2}=1.298$.

The controlled operator $U_{1}$ can be obtained as $R_{y}\left(1.298\right)$. At this point, we can give the quantum circuit for each iteration of the solution Equation (40), as shown in Figure 6

**Figure 7:**
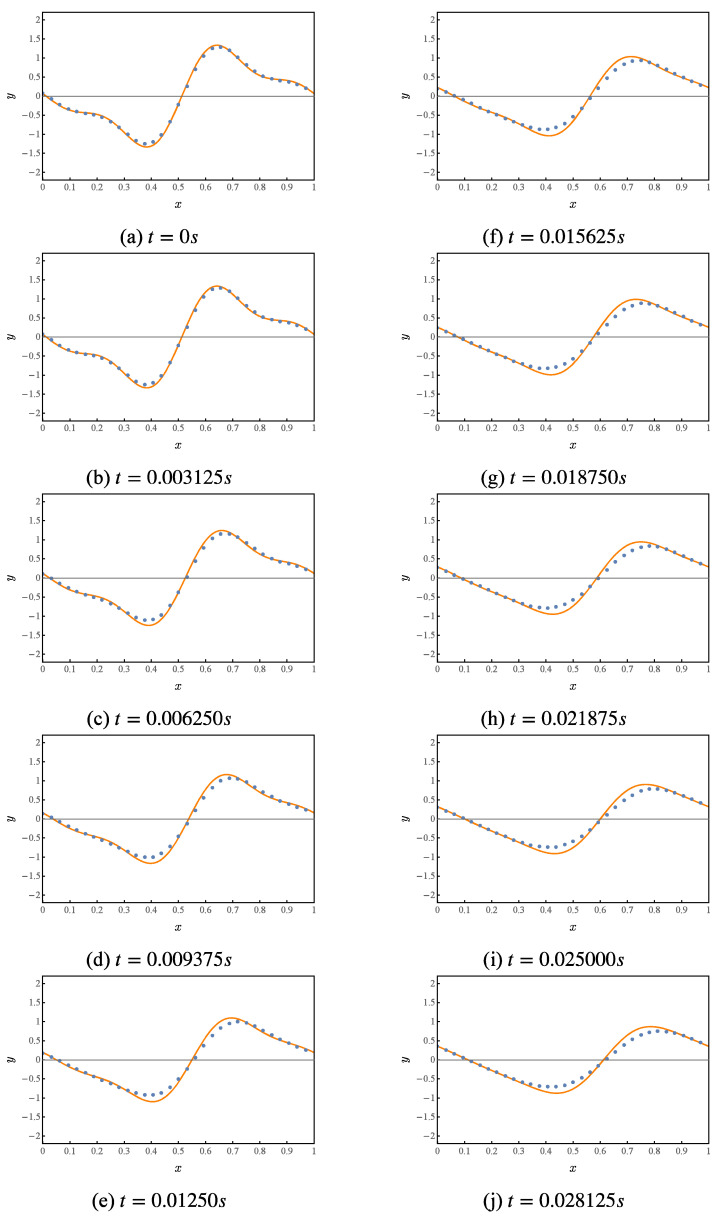
Numerical simulation of the quantum solution algorithm for the traveling wave dissipation problem of the first-order wave equation.

Numerical simulation of the first 10 iterations of this quantum circuit results in Figure 7. The orange curve represents the resolved theoretical value. The blue points represent the values solved by the numerical simulation of the quantum algorithm.

## 4. Duality Quantum Algorithm for Solving Traveling Wave Dispersion Problems

By adding the dispersion term to Equation (10), the traveling wave equation with dispersion is obtained as

(44) $$\frac{\partial u}{\partial t}+k\frac{\partial u}{\partial x}+\beta \frac{\partial^{3}u}{\partial x^{3}}=0.$$

Taylor expansion of the terms of Equation (44)

(45) $$\begin{array}{cc} \; & \frac{\partial u}{\partial t}=\frac{u\left(x,t+\tau \right)-u\left(x,t\right)}{\tau }+o\left(\tau \right),\\ \; & \frac{\partial u}{\partial x}=\frac{u\left(x+h,t\right)-u\left(x,t\right)}{h}+o\left(h\right),\\ \; & \frac{\partial^{3}u}{\partial x^{3}}=\frac{-u(x-2h,t)+3u(x-h,t)-3u(x,t)+u(x+h,t)}{h^{3}}+o\left(h^{3}\right). \end{array}$$

Differentiating Equation (44) yields

(46) $$\begin{array}{cc} \; & \frac{u\left(x_{i},t_{j+1}\right)-u\left(x_{i},t_{j}\right)}{\tau }+k\frac{u\left(x_{i+1},t_{j}\right)-u\left(x_{i},t_{j}\right)}{h}\\ \; & \;\;+\beta \frac{-u\left(x_{i-2},t_{j}\right)+3u\left(x_{i-1},t_{j}\right)-3u\left(x_{i},t_{j}\right)+u\left(x_{i+1},t_{j}\right)}{h^{3}}=0. \end{array}$$

Let $\Delta =\frac{\tau }{h}$, $\Delta_{2}=\beta \frac{\Delta }{h^{2}}$, and the following iterative relation can be obtained

(47) $$\begin{array}{cc} u\left(x_{i},t_{j+1}\right)=\Delta_{2}u\left(x_{i-2},t_{j}\right)- & 3\Delta_{2}u\left(x_{i-1},t_{j}\right)\\ \; & +\left(k\Delta +3\Delta_{2}+1\right)u\left(x_{i},t_{j}\right)-\left(k\Delta +\Delta_{2}\right)u\left(x_{i+1},t_{j}\right). \end{array}$$

Take the periodic boundary condition that $u\left(x_{N},t\right)=u\left(x_{0},t\right)$. Then the equation describing the whole system can be written in the following form

(48) $$\left[\begin{array}{c} u\left(x_{0},t_{j+1}\right)\\ u\left(x_{1},t_{j+1}\right)\\ \ldots \\ u\left(x_{N-1},t_{j+1}\right) \end{array}\right]=A\left[\begin{array}{c} u\left(x_{0},t_{j}\right)\\ u\left(x_{1},t_{j}\right)\\ \ldots \\ u\left(x_{N-1},t_{j}\right) \end{array}\right],$$

where

(49) $$A=\left[\begin{array}{ccccccccccccc} a & b & 0 & \; & & \; & \ldots & \; & \ldots & \; & 0 & d & c\\ c & a & b & 0 & \; & & \ldots & \; & \ldots & \; & & 0 & d\\ d & c & a & b & 0 & \; & \ldots & \; & \ldots & \; & & \; & 0\\ 0 & d & c & a & b & 0 & \ldots & \; & \ldots & \; & & \; & 0\\ \; & \; & \ldots & \; & & \; & \ldots & \; & \ldots & \; & & \; & \\ 0 & \; & & \ldots & \; & & \ldots & 0 & d & c & a & b & 0\\ 0 & \; & & \ldots & \; & & \ldots & \; & 0 & d & c & a & b\\ b & 0 & \; & \ldots & \; & & \ldots & \; & & 0 & d & c & a \end{array}\right],$$

in which

(50) $$\begin{array}{cc} \; & a=k\Delta +3\Delta_{2}+1,\\ \; & b=-\left(k\Delta +\Delta_{2}\right),\\ \; & c=-3\Delta_{2},\\ \; & d=\Delta_{2}. \end{array}$$

It can be seen that $A=\left(k\Delta +3\Delta_{2}+1\right)A_{0}-\left(k\Delta +\Delta_{2}\right)A_{1}-3\Delta_{2}A_{2}+\Delta_{2}A_{3}$, where

(51) $$A_{3}={\left[\begin{array}{ccccccccc} 0 & 0 & \; & & \; & 0 & 0 & 1 & 0\\ 0 & 0 & \; & & \; & 0 & 0 & 0 & 1\\ 1 & 0 & \; & & \ldots & 0 & 0 & 0 & 0\\ 0 & 1 & \; & & \; & 0 & 0 & 0 & 0\\ \; & \; & \ldots & \; & & \; & & \; & \\ \; & \ldots & \; & 1 & 0 & 0 & 0 & 0 & 0\\ \; & \; & & 0 & 1 & 0 & 0 & 0 & 0\\ 0 & 0 & \; & 0 & 0 & 1 & 0 & 0 & 0\\ 0 & 0 & \; & 0 & 0 & 0 & 1 & 0 & 0 \end{array}\right]}_{N⊗N}.$$

**Figure 8:**
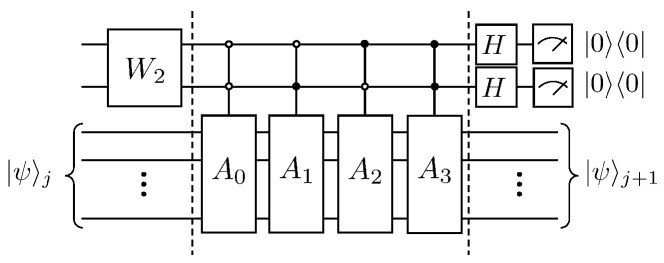
Quantum circuit for solving the dispersion problem of the first-order wave equation.

Therefore, the operation of the linear combination of unitary operators can be equivalently implemented by introducing two auxiliary qubits, whose quantum circuit is shown in Figure 8.

It is not difficult to find $A_{3}=A_{2}^{2}$, and the following will be combined with the quantum circuit (Figure 8) to explain the duality quantum algorithm for the solution of the dispersion problem of the first-order wave equation. First, the auxiliary qubits pass through the $W_{2}$ gate, the effect of which is as follows

(52) $$W_{2}:|00\rangle \to \frac{\left(k\Delta +3\Delta_{2}+1\right)|00\rangle +\left(k\Delta +\Delta_{2}\right)|01\rangle -3\Delta_{2}|10\rangle +\Delta_{2}|11\rangle }{\sqrt{{\left(k\Delta +3\Delta_{2}+1\right)}^{2}+{\left(k\Delta +\Delta_{2}\right)}^{2}+10\Delta_{2}^{2}}}.$$

Next, after four controlled quantum gates, $\left|00\rangle \langle 00\right|⊗A_{0}$, $\left|01\rangle \langle 01\right|⊗A_{1}$, $\left|10\rangle \langle 10\right|⊗A_{2}$ and $\left|11\rangle \langle 11\right|⊗A_{3}$, the quantum state evolves as

(53) $$\frac{\left(k\Delta +3\Delta_{2}+1\right)|00\rangle A_{0}{|\psi \rangle }_{j}+\left(k\Delta +\Delta_{2}\right)|01\rangle A_{1}{|\psi \rangle }_{j}-3\Delta_{2}|10\rangle A_{2}{|\psi \rangle }_{j}+\Delta_{2}|11\rangle A_{3}{|\psi \rangle }_{j}}{\sqrt{{\left(k\Delta +3\Delta_{2}+1\right)}^{2}+{\left(k\Delta +\Delta_{2}\right)}^{2}+10\Delta_{2}^{2}}}.$$

Then, the Hadamard transform is performed for the two auxiliary qubits, and the quantum state evolves as

(54) $$\begin{array}{cc} \frac{1}{2} & |00\rangle \left[\frac{\left(k\Delta +3\Delta_{2}+1\right)A_{0}{|\psi \rangle }_{j}+\left(k\Delta +\Delta_{2}\right)A_{1}{|\psi \rangle }_{j}-3\Delta_{2}A_{2}{|\psi \rangle }_{j}+\Delta_{2}A_{3}{|\psi \rangle }_{j}}{\sqrt{{\left(k\Delta +3\Delta_{2}+1\right)}^{2}+{\left(k\Delta +\Delta_{2}\right)}^{2}+10\Delta_{2}^{2}}}\right]\\ + & \frac{1}{2}|01\rangle \left[\frac{\left(k\Delta +3\Delta_{2}+1\right)A_{0}{|\psi \rangle }_{j}-\left(k\Delta +\Delta_{2}\right)A_{1}{|\psi \rangle }_{j}-3\Delta_{2}A_{2}{|\psi \rangle }_{j}-\Delta_{2}A_{3}{|\psi \rangle }_{j}}{\sqrt{{\left(k\Delta +3\Delta_{2}+1\right)}^{2}+{\left(k\Delta +\Delta_{2}\right)}^{2}+10\Delta_{2}^{2}}}\right]\\ + & \frac{1}{2}|10\rangle \left[\frac{\left(k\Delta +3\Delta_{2}+1\right)A_{0}{|\psi \rangle }_{j}+\left(k\Delta +\Delta_{2}\right)A_{1}{|\psi \rangle }_{j}+3\Delta_{2}A_{2}{|\psi \rangle }_{j}-\Delta_{2}A_{3}{|\psi \rangle }_{j}}{\sqrt{{\left(k\Delta +3\Delta_{2}+1\right)}^{2}+{\left(k\Delta +\Delta_{2}\right)}^{2}+10\Delta_{2}^{2}}}\right]\\ + & \frac{1}{2}|11\rangle \left[\frac{\left(k\Delta +3\Delta_{2}+1\right)A_{0}{|\psi \rangle }_{j}-\left(k\Delta +\Delta_{2}\right)A_{1}{|\psi \rangle }_{j}+3\Delta_{2}A_{2}{|\psi \rangle }_{j}+\Delta_{2}A_{3}{|\psi \rangle }_{j}}{\sqrt{{\left(k\Delta +3\Delta_{2}+1\right)}^{2}+{\left(k\Delta +\Delta_{2}\right)}^{2}+10\Delta_{2}^{2}}}\right]. \end{array}$$

Finally, the state of the auxiliary qubit is selected as 00 after measurement, then the state of the working qubits at this time is ${|\psi \rangle }_{j+1}$, that is

(55) $$\frac{\left(k\Delta +3\Delta_{2}+1\right)A_{0}{|\psi \rangle }_{j}+\left(k\Delta +\Delta_{2}\right)A_{1}{|\psi \rangle }_{j}-3\Delta_{2}A_{2}{|\psi \rangle }_{j}+\Delta_{2}A_{3}{|\psi \rangle }_{j}}{\sqrt{{\left(k\Delta +3\Delta_{2}+1\right)}^{2}+{\left(k\Delta +\Delta_{2}\right)}^{2}+10\Delta_{2}^{2}}}.$$

Define the coefficient $C_{j}$ as

(56) $$\sqrt{\sum_{i=0}^{N-1}u^{2}\left(x_{i},t_{j}\right)\left[{\left(k\Delta +3\Delta_{2}+1\right)}^{2}+{\left(k\Delta +\Delta_{2}\right)}^{2}+10\Delta_{2}^{2}\right]}.$$

The amplitude under the computational basis of the quantum state ${|\psi \rangle }_{j+1}$ is enlarged $C_{j}$ times to obtain the column vector $u\left(x_{i},t_{j+1}\right)$, which is the state of the system at the moment $t_{j+1}$. According to Lemma 5.5 and Lemma 7.1 in article [24], and combined with the analysis of Figure 8, we can obtain that the computational complexity of this algorithm for each iteration is $O\left(N\right)$, while the complexity of the classical algorithm is $O\left(N^{2}\right)$. The specific calculation of the complexity is presented at the end of this paper.

The following equation will be used as an example

(57) $$\left\{\begin{array}{c} \frac{\partial u}{\partial t}+2\frac{\partial u}{\partial x}+0.01\frac{\partial^{3}u}{\partial x^{3}}=0\\ u\left(x,0\right)=-\sin2\pi x+\frac{\sin\;4\pi x}{2}-\frac{\sin\;6\pi x}{3} \end{array}\right.$$

to show the duality quantum algorithm for the solution of the dispersion problem of the first-order wave equation. Firstly, one period of the function is chosen, i.e., [0,1], and this interval is discretized into 32 points, i.e., h=0.03125 in Equation (47). Thus, the function value of 32 discrete points can be encoded using 5 qubits. Choose Δ=0.05 and $\Delta_{2}=0.512$ in Equation (47). Then τ=0.0015625, which represents the time interval for each iteration of the system evolution. According to Equation (52), the effect of the action of $W_{2}$ can be determined as

(58) $$W_{2}:|00\rangle \to 0.8359|00\rangle +0.1941|01\rangle -0.4871|10\rangle +0.1624|11\rangle .$$

It is constructed as shown in Figure A1 with Equations (A1) and (A2). Construct the revolving gate $R_{n}\left(\theta \right)$ of the first auxiliary qubit according to Equation (A1), such that

(59) $$R_{n}\left(\theta \right)|00\rangle \to \left(0.8581|0\rangle +0.5135|1\rangle \right)|0\rangle .$$

It is obtained that $R_{n}\left(\theta \right)$ is $R_{y}\left(1.078\right)$.

According to Equation (A3), two controlled operators $U_{1}$, $U_{2}$ are to be realized as follows

(60) $$\begin{array}{cc} U_{1} & |0\rangle \to 0.9741|0\rangle +0.2262|1\rangle ,\\ U_{2} & |0\rangle \to -0.9486|0\rangle +0.3163|1\rangle . \end{array}$$

**Figure 9:**
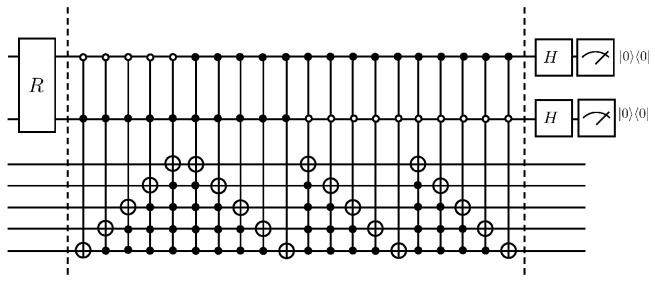
Example of a quantum circuit for solving the dispersion problem of the first-order wave.

The controlled operator $U_{1}$ can be obtained as $R_{y}\left(0.4563\right)$ and $U_{2}$ as $R_{y}\left(5.639\right)$. The quantum circuit for each iteration of the solution Equation (57) so far is given in Figure 9.

**Figure 10:**
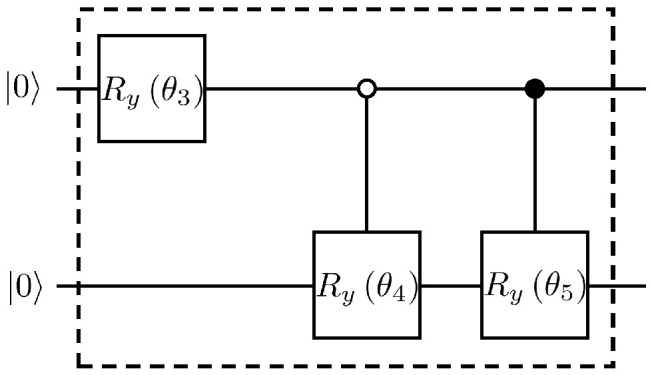
The specific quantum circuit of the *R*-operation, where $\theta_{3}=1.078$, $\theta_{4}=0.4563$, $\theta_{5}=5.639$.

And the specific quantum circuit of the *R*-operation in Figure 9 is shown in Figure 10.

**Figure 11:**
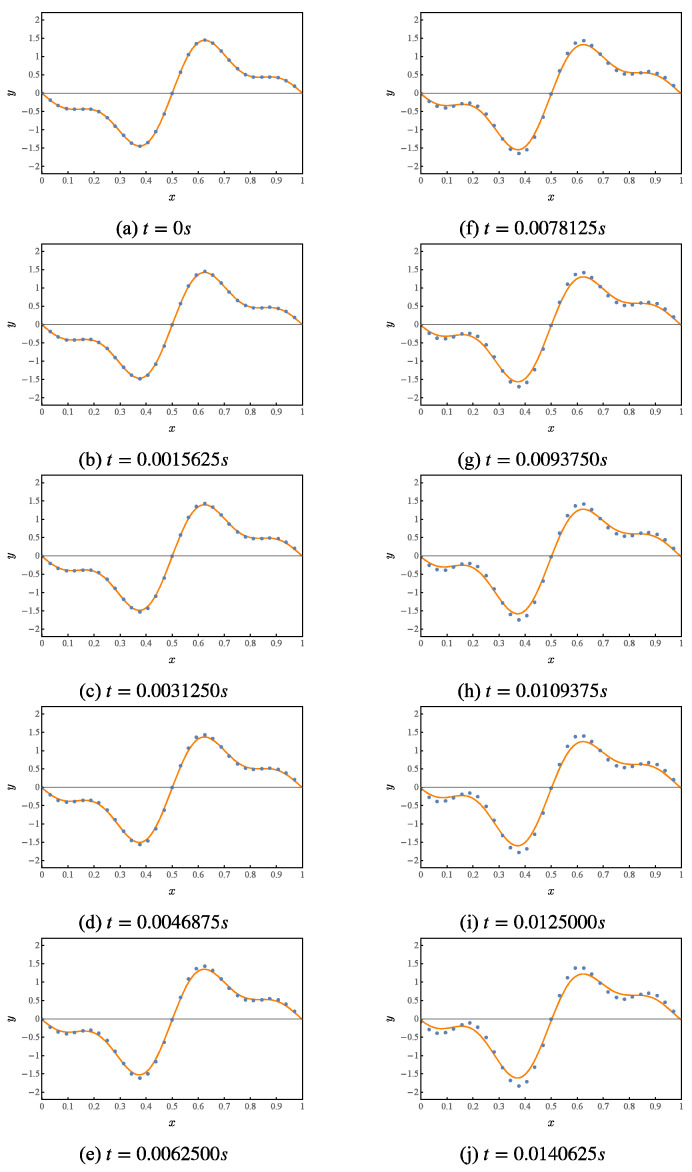
Numerical simulation of a quantum solution algorithm for the dispersion problem of the first-order wave equation.

The result of numerically simulating the first 10 iterations of this quantum circuit is shown in Figure 11. The orange curve represents the theoretical value. The blue points represent the values solved by the numerical simulation quantum algorithm.

## 5. Discussion

For a quantum algorithm that solves a *d*-dimensional partial differential equation (meaning that there are *d* spatial variables) with a spatial discretization number of *N*, the output is an approximation C(f) of the function *f* with an error ϵ. In fact, for the problem of quantum algorithms solving partial differential equations, the number of discrete points *N* and the error ϵ are interrelated [12,25]. The correlations are as follows

(61) $$N=O\left(\mathrm{poly}\left(\frac{1}{\epsilon^{d}}\right)\right).$$

For the preparation of the initial state, its complexity is $O\left(\mathrm{poly}\log\left(N\right)\right)$. The complexity of each iteration of the algorithm in this paper will be given below. First, according to Lemma 5.5 and Lemma 7.1 in article [24], it can be obtained that the controlled gate $C^{n-1}\left(U\right)$ for *n* qubits having n−1 control qubits can be split into CNOT gates and single-qubit gates for a total of $2^{n+1}-5$, where n≥3. For the quantum circuit in Figure 1, the total number of elementary quantum gates required is

(62) $$\begin{array}{c} 3+O\left(C^{2}\left(U\right)\right)+\cdots +O\left(C^{n}\left(U\right)\right)\\ =2^{n+3}-5n-8=8N-5\log_{2}N-8≃O\left(N\right) \end{array}$$

For the quantum circuit in Figure 5, the total number of elementary quantum gates required is

(63) $$\begin{array}{c} 8+2\left[O\left(C^{2}\left(U\right)\right)+\cdots +O\left(C^{n+1}\left(U\right)\right)\right]\\ =2^{n+5}-10n-24=32N-10\log_{2}N-24≃O\left(N\right) \end{array}$$

For the quantum circuit in Figure 8, the total number of elementary quantum gates required is

(64) $$\begin{array}{c} 8+4\left[O\left(C^{2}\left(U\right)\right)+\cdots +O\left(C^{n+1}\left(U\right)\right)\right]\\ =2^{n+6}-20n-56=64N-20\log_{2}N-56≃O\left(N\right) \end{array}$$

It can be found that the quantum-solving algorithm given in this paper has a quadratic acceleration for each iteration compared to the classical algorithm. However, the state of the auxiliary qubits needs to be selected after measurement at the end of each iteration. This result is probabilistic, and the overall success rate of the algorithm decreases exponentially as the number of iterations increases if the selection is made after each iteration. Therefore, ensuring an overall higher success rate requires the use of the quantum search algorithm [17,18,19,20] to amplify the amplitude of the target state before measurement. Under ideal conditions of the device, it is proved that the Grover–Long algorithm can achieve a 100% success rate in all cases [17,26,27]. Thus, using the Grover–Long algorithm under ideal conditions to amplify the amplitude, it is possible to obtain a 100% success rate every time. If the complexity of each iteration step is considered synthetically, then the complexity of each iteration step is $O\left(N\right)+O\left(\sqrt{M}\right)$, where *M* is the dimension of the auxiliary qubits space.

In the future, our quantum algorithms are expected to be combined with finite element methods to solve complex practical problems, such as those related to fluid dynamics [28].

## Data Availability

Not applicable.

## Appendix A

The quantum circuit for the preparation of two-qubit arbitrary quantum states is as follows in Figure A1.

First, the rotation operator $R_{n}\left(\theta \right)$ is constructed, such that

(A1) $$|00\rangle \to \left(\sqrt{c_{0}^{2}+c_{1}^{2}}|0\rangle +\sqrt{c_{2}^{2}+c_{3}^{2}}|1\rangle \right)|0\rangle .$$

In the second step, construct the controlled quantum gates $U_{1}$ and $U_{2}$, such that

(A2) $$\begin{array}{cc} \sqrt{c_{0}^{2}+c_{1}^{2}} & |0\rangle \left(\frac{c_{0}}{\sqrt{c_{0}^{2}+c_{1}^{2}}}|0\rangle +\frac{c_{1}}{\sqrt{c_{0}^{2}+c_{1}^{2}}}|1\rangle \right)\\ + & \sqrt{c_{2}^{2}+c_{3}^{2}}|1\rangle \left(\frac{c_{2}}{\sqrt{c_{2}^{2}+c_{3}^{2}}}|0\rangle +\frac{c_{3}}{\sqrt{c_{2}^{2}+c_{3}^{2}}}|1\rangle \right), \end{array}$$

where

(A3) $$\begin{array}{cc} U_{1}|0\rangle & \to \frac{c_{0}}{\sqrt{c_{0}^{2}+c_{1}^{2}}}|0\rangle +\frac{c_{1}}{\sqrt{c_{0}^{2}+c_{1}^{2}}}|1\rangle ,\\ U_{2}|0\rangle & \to \frac{c_{2}}{\sqrt{c_{2}^{2}+c_{3}^{2}}}|0\rangle +\frac{c_{3}}{\sqrt{c_{2}^{2}+c_{3}^{2}}}|1\rangle . \end{array}$$
